# Social accountability for maternal health services in Muanda and Bolenge Health Zones, Democratic Republic of Congo: a situation analysis

**DOI:** 10.1186/s12913-015-1176-6

**Published:** 2015-11-23

**Authors:** Eric M. Mafuta, Marjolein A. Dieleman, Lisanne M. Hogema, Paul N. Khomba, François M. Zioko, Patrick K. Kayembe, Tjard de Cock Buning, Thérèse N. M. Mambu

**Affiliations:** Kinshasa School of Public Health, Faculty of Medicine, University of Kinshasa, Po Box: 11850, Kinshasa, DR Congo; Royal Tropical Institute, Amsterdam, The Netherlands; Athena Institute, Faculty of Life Sciences, VU University, Amsterdam, The Netherlands; Cordaid Representative Office, Kinshasa, DR Congo; Medicus Mundi Representative Office, Kinshasa, DR Congo; Kinshasa School of Public Health, Faculty of Medicine, University of Kinshasa, Po Box: 11850, Kinshasa, DR Congo

**Keywords:** Governance, Social accountability, Community participation, Maternal health, Women, Democratic Republic of the Congo

## Abstract

**Background:**

The Democratic Republic of the Congo is one of the countries in Sub-Saharan Africa with the highest maternal mortality ratio estimated at 846 deaths per 100,000 live births. Innovative strategies such as social accountability are needed to improve both health service delivery and utilization. Indeed, social accountability is a form of citizen engagement defined as the ‘extent and capability of citizens to hold politicians, policy makers and providers accountable and make them responsive to their needs.’ This study explores existing social accountability mechanisms through which women’s concerns are expressed and responded to by health providers in local settings.

**Methods:**

An exploratory study was conducted in two health zones with purposively sampled respondents including twenty-five women, five men, five health providers, two health zone officers and eleven community stakeholders. Data on women’s voice and oversight and health providers’ responsiveness were collected using semi-structured interviews and analysed using thematic analysis.

**Results:**

In the two health zones, women rarely voiced their concerns and expectations about health services. This reluctance was due to: the absence of procedures to express them, to the lack of knowledge thereof, fear of reprisals, of being misunderstood as well as factors such as age-related power, ethnicity backgrounds, and women’s status.

The means most often mentioned by women for expressing their concerns were as individuals rather than as a collective. They did not use them instead; instead they looked to intermediaries, mostly, trusted health providers, community health workers and local leaders. Their perceptions of health providers’ responsiveness varied. For women, there were no mechanisms for oversight in place. Individual discontent with malpractice was not shown to health providers. In contrast, health providers mentioned community health workers, health committee, and community based organizations as formal oversight mechanisms. All respondents recognized the lack of coalition around maternal health despite the many local associations and groups.

**Conclusions:**

Social accountability is relatively inexistent in the maternal health services in the two health zones. For social accountability to be promoted, efforts need to be made to create its mechanisms and to open the local context settings to dialogue, which appears structurally absent.

**Electronic supplementary material:**

The online version of this article (doi:10.1186/s12913-015-1176-6) contains supplementary material, which is available to authorized users.

## Background

Maternal mortality remains a major health issue in developing countries such as the Democratic Republic of the Congo (DRC) [1]. A recent survey estimated the maternal mortality ratio at 846 deaths per 100,000 live births [2], indicating that the DRC has not reached the MDG 5’s target [2, 3]. Interventions to reduce maternal morbidity and mortality emphasize the health service utilization through facility-based childbirth and skilled attendance at birth with timely referral for emergency obstetric care if complications occur [4, 5].

Skilled providers, appropriate equipment and services are important but there is no guarantee for responsive services. Service quality as perceived by its patients can improve the health service utilization by changing the behaviour of healthcare providers towards their patients and by improving their responsiveness to needs and expectations of patients [6–9]. One way of assessing and improving the behaviour of providers towards patients is through the use of social accountability mechanisms [10, 11].

Social accountability is defined as, ‘accountability that relies on civic engagement, i.e. in which citizens and/or civil society organizations participate directly or indirectly in exerting accountability’ [12] and holding politicians, policy makers and service providers responsible for their performance [13–15]. Functioning social accountability mechanisms should result in responsive services, defined as changes made to the service on the basis of ideas or concerns raised by users [16, 17]. Responsiveness also corresponds to the capacity of the service to limit abusive behaviour or inappropriate treatment by providers as well as to mitigate the fears and the shame which are associated with problems [13]. In the health sector, a responsive health service favours health by impacting the choice of persons and encouraging the use of health care by the population [18]. It is argued that, under certain conditions, social accountability mechanisms can trigger the responsiveness of health service providers and policymakers. Increased responsiveness is ultimately expected to result in a stronger health, such as an increase in user satisfaction or service utilization, or a decrease in the prevalence of disease, in our case maternal mortality.

Some examples of successful community participation projects with accountability mechanisms come from rural Nepal and rural Cambodia, where respectively Manandhar et al. (2004) and Skinner and Rathavy (2009) showed that when the citizens, are empowered to express their views and discuss the quality of health facility performance, when their views are taken into account in the decision making process, that could contribute to change, i.e. maternal health services could be adapted to their needs and might contribute better to the reduction of maternal mortality [19, 20]. In Nepal, for example, at the end of the project, the maternal mortality ratio was about 80 % lower within the intervention areas compared to the controls clusters [19]. Involving citizens could be an important strategy to improve the relationship between providers and clients particularly in fragile states, which are characterized by weak government systems and poor health indicators [16]. To date, we have not identified studies exploring social accountability for maternal health services performance and responsiveness in the DRC.

This paper presents some of the perceived realities of the current situation regarding social accountability in maternal health services in one health zone in the province of Bas-Congo and one health zone in the province of Equateur in the DRC.

## Methods

### Research question and conceptual framework

We aimed at answering the following question: What mechanisms and experiences exist regarding social accountability in maternal health services in Bas-Congo and Equateur?

**Fig. 1:**
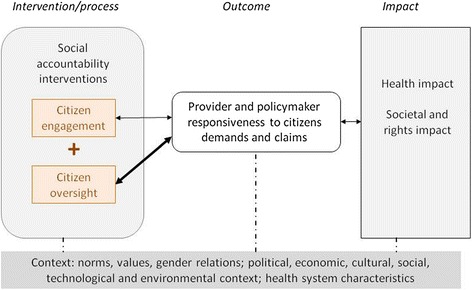
Conceptual framework for social accountability (Lodenstein et al., 2013)

In order to answer this question, we implemented an exploratory study in two Health Zones using the conceptual framework of social accountability proposed by Lodenstein et al. [21], refer to Fig. 1. This model distinguishes three elements in a social accountability mechanism:

1. Citizen engagement, includes individual participation and voice or collective expression of one’s expectations and concerns without formal ways of enforcement;
2. Citizen oversight, which includes involving citizens in collective monitoring and evaluation of health services and the performance of health service providers, sanctioning when the poor performance occurs and rewarding when the performance is perceived as of quality;
3. Both might result in a higher degree of responsive health services, thus contributing to improve health.

Social accountability mechanisms, responsive behaviour, and their consequences are influenced by contextual factors, such as societal values, gender relations, levels of political stability and health system characteristics. In the current paper, we explore only elements of social accountability mechanisms without taking into account contextual factors.

### Study setting

The DRC is divided into 516 Health Zones (HZs), and each health zone has 10–20 health areas. Each health area consists of several villages and is serviced by a health centre. A functioning health centre is a health facility that provides an essential healthcare package comprising of among other activities, under five years’ growth and development screening and maternal health care to approximately 5,000 people in rural settings and to 15,000 in urban settings [22]. Health centres normally charge user fees. Each health centre should have a health committee that is in charge of managing health centre resources, planning and monitoring activities of the centre, identifying population’s health needs, and organizing community-based health activities by community health workers. The health committee consists of about 10 members and includes health providers, representatives of community health workers, and elected community leaders [22, 23]. Every month, Community Health Workers (CHWs), who are in principle in charge of visiting 15–25 households in a village, submit a report to their representatives at the health committee. The latter then present a summary at the health committee meeting and receive from health providers a report on the health centre’s performance. A functioning health committee carries out its planned activities, meets monthly to discuss community health issues and reports them to the Health Zone management team [24].

It is within two of these health zones that the study was carried out: Muanda and Bolenge. They were selected on the basis of the following criteria: a rural zone, in which an organization implements (or intends to implement) an intervention with a social accountability component and that would be interested in having research-action added to their programme. Muanda HZ is situated in Bas Congo, one of the richest provinces of the DRC, at the crossroads of trading routes between Angola, Congo Brazzaville and the DRC [25]. Since 2008, it has benefited from a performance based financing (PBF) project aimed at improving health services supported by Cordaid, an international NGO. Bolenge HZ is situated in Equateur, one of the poorest provinces in the DRC [26]. Bolenge benefits from a community-based health insurance scheme supported by Medicus Mundi. All performance based financing and community based health insurance scheme have a social accountability component. Table 1 provides an overview of these health zones. In each health zone, the main selection criterion for one health area to be purposively selected was for its functioning health centre and a functioning health committee.

**Table 1:** Essential contexts indicators of selected study health zones

Indicators	Muanda	Bolenge
Population	137,178	79,648
Number of health centers	9	15
Number of referral health facilities	2	1
Health facility attendance rate (%)	43.8	46.5
Antenatal health care attendance rate	98.0	91.3
Proportion of pregnant women with more 4 visits and more	46.2	40.2
Health providers’ attendance at birth rate (%)	95.1	78.4
Main population occupations	Agriculture	Agriculture
	Fishery	Fishery
	Small trade	Small trade
	Oil Firm employment	
Population composition	Bantu ethnic groups	Bantu ethnic groups Pygmies
Existence of basic services		
Safe water supply	Yes	No
Electric power supply	Yes	No

### Study population and sampling

Within the selected Health Areas, two different groups were included as respondents. The first group comprises of community members: women within the reproductive age (between 15 and 49), with a child or expecting a child or with a history of recent pregnancy complications, men and their mothers-in-law. For maximum variation purposes, three different age groups were established among women : ≤ 19 years; 20–35 years and >35 years according to risk for pregnancies related issues [27, 28]. In each health area at least three women in each age category, two men, and two mothers-in-law were included, until no new information emerged from interviews.

The second group consisted of health providers: the nurse in charge of the health centre and a regular nurse, responsible for the provision of maternal health services.

In addition, key informants were interviewed: the health zone chief officer (HZCO), the health project manager, the political administrative authority, a women association representative, health committee member, a community health worker and a traditional birth attendant. They were selected according to their influence in the community, and their understanding of women’s needs.

### Data collection and issues

Community members, health providers and key informants were interviewed between September and October 2013 by means of individual in-depth semi-structured, audiotaped interviews with an interview guide on; the specific content areas based on the aim of the study, the current literature on social accountability and other topics addressing similar aspects. These areas were women’s expectations, needs and concerns regarding maternal health services, as well as (in) formal ways to voice and express their concerns. Also explored was citizen oversight, identification of procedures in place for women to monitor and evaluate health providers’ performance and to reward or sanction health providers. The researchers also explored the perceived health providers’ responsiveness (refer to Additional file 1).

Community members were approached outside of their homes and invited to participate in this research. A community health worker was consulted to assist in identifying community members that could be invited and to whom interviews could be submitted. When their consent was provided, the interview took place in their homes. Health providers and most key informants were approached at their workplace. The interviews took place after work, once they agreed to participate and their consent was provided. Interviews consisted of approximately 45-min audio recordings were conducted in Lingala or in French. The field team consisted of three researchers; firstly an author, a research assistant from the Athena Institute/VU Amsterdam, and a research assistant from the University of Kinshasa, supervised by a senior researcher. The CHW who guided researchers did not participate in the interview and was excluded as a respondent. Interviews with women and mothers-in-law were conducted by female research assistants. On the other hand, interviews with men, health providers and key informants were carried out by the first author. A debriefing session was held after each fieldwork day during which themes, impressions of the findings and procedures were discussed and documented in field notes.

### Data processing and analysis

Interviews were transcribed *verbatim* in Lingala, translated into French and checked by two team members. Atlas-ti 6.1 software (ATLAS.ti GmbH, Berlin) was used to organize the qualitative data. Both inductive and deductive approaches of thematic content analysis were used. The analysis was performed in two main stages. During the first stage, each transcript was separately read, identifying themes that emerge based on the SA framework and research questions, but when a new theme emerged, it was included. At this stage, transcripts were read repeatedly so as to become familiar with the participants’ stories and to identify themes in each of the separate interviews. These first impressions, thoughts and initial analysis were recorded. During the second stage, the researchers examined themes that had arisen in the initial analysis of each separate interview, seeking connections, similarities and differences. Four steps were taken to enhance the credibility of the study - the research team received training in interview techniques, the interview guides were pre-tested and adapted accordingly and the interview guides were written in French, translated into local languages and translated back into French. The results and interpretations were critically discussed by the research team and with local health partners.

### Ethical considerations

The study was approved by the Institutional Review Board, Kinshasa School of Public Health and all research procedures were in accordance with the Helsinki Declaration. Most participants provided written consent, and four community members provided oral consent prior to interviews. To protect interviewees’ anonymity, only category type and research site were used.

## Results

The results’ section commences with a description of the respondents; thereafter research results are presented on expectations, voice, oversight and perceived responsiveness.

### Participants

In total, 48 interviews were conducted in the two selected areas, viz., 27 in Bolenge HZ, and 21 in Muanda HZ. Table 2 shows an overview of the participants who were interviewed. Since the focus is on maternal health, women in their reproductive period presented the largest group of respondents (*n* = 21). Their ages ranged from 17–39 years (median: 27 years). The median number of children per woman was three offspring with ages ranging from two weeks to six years; most of them were from Bantu tribes except in Bolenge where some Pygmies reside. Women were mostly farmers, with a primary school education and lived with a partner. Four women had experienced complications during their pregnancy, and one had miscarried (Tables 3 and 4).

**Table 2:** Respondents’ categories

Respondents categories	Bolenge	Muanda	Total
Health providers			
Medical Health Officers	1	1	2
Head Nurses	2	1	3
Maternal health nurses	1	1	2
Community Key Informants			
Community association president	1	0	1
Women association presidents	1	1	2
Health committee president	1	0	1
Community health workers	1	1	2
Health Project managers	1	1	2
Local authority	2	1	3
Community members			
Men	2	3	5
Women	11	9	20
Mother in law	3	2	5
Total	27	21	48

**Table 3:** Women characteristics (Muanda)

Muanda – Women (Age range 17–39)						
Interviewee	Age	Number of children	Last child’s age	ANC	Occupation	Birthing place
001	28	2	2 months	Yes	Hair styler	Muanda city HC
002	39	6	4 months	Yes	Saleswoman/nurse	Muanda GRH
003	18	1	2 weeks	Yes	Student	Hygiene HC
004	23	1	1 month ½	Yes	Housewife/Farmer	Nsiamfumu HC
005	27	3	5 years	Yes	Housewife/saleswoman	Muanda GRH (^a^)
006	33	6	2 months	Yes	Housewife / Teacher	Muanda GRH (^a^)
007	28	4	1 months ½	Yes	Saleswoman	Nsiamfumu HC
008	17	0	-	Yes	Student	Pregnant (^a^)
009	17	0	-	Yes	Student	Pregnant (^a^)

(^a^) History of complications HC: Health centre GRH: General Referral Hospital ANC: Antenatal care

**Table 4:** Women’s characteristics (Bolenge)

Bolenge – Women (Age range 17–37)						
Interviewee	Age	Number of children	Last child’s age	ANC	Occupation	Birthing place
001	37	4	6 years	Yes	Housewife/Farmer	Bolenge GRH (^a^)
002	31	7	4 days	Yes	Housewife/Farmer	Iyonda HC
003	27	3	2 months	Yes	Housewife/Farmer	Iyonda HC
004	33	1	12 days	Yes	Teacher	Pregnant (^a^)
005	25	2	2 months	Yes	Housewife/farmer	On the road to Iyonda (P)
006	18	1	5 days	Yes	Student	Iyonda HC
007	28	4	1 month ½	Yes	Housewife/Farmer	Iyonda HC
008	17	0	-	Yes	Student	Pregnant
009	23	4	2 months	Yes	Housewife/farmer	Iyonda HC
010	27	4	4 days	Yes	Housewife/farmer	Iyonda HC (P)
011	22	2 (1D)	2 weeks	Yes	Housewife/farmer	Iyonda HC

(D) Deceased (^a^) History of complications (P) Pygmy HC: Health centre GRH: General Referral Hospital ANC: Antenatal care

### Women’s concerns and complaints regarding health services

The term ‘voice’ comprises of five aspects in English: *speaking up* with respect to *needs*, *expectations* and *concerns* regarding healthcare facilities. It also comprises of *complaints* about healthcare services. In Lingala these different aspects of the word ‘voice’ are translated by the word *posa*, which literally means ‘concerns.’ In the text, the word ‘concern’ is used, and therefore, also refers to expectations and needs. However, to what extent these are actually voiced, in the sense of speaking out, will be reported in the section entitled ‘women’s voice.’

As a matter of fact, the researchers dealt with women’s concerns and complaints regarding maternal health services. At both sites, most women sounded positive about the health care provided, they did not complain about it and were unsure on what to ask. Most women perceived healthcare providers (HPs) as professionals who have the required skills to cure/help them. They believed that they had less knowledge than HPs because they considered themselves laypersons unable to judge how healthcare should be provided. They were unsure on how to converse and make their concerns known to HPs, as portrayed in the following quotation:

“When I have a problem related to my health or my pregnancy, I go to the HP and tell him all I noticed and felt. He will provide me with healthcare. I don’t see another way to proceed. I follow only what the nurse tells me to do. What he says is good for my health. It is up to him to direct my healthcare process.”

*(Woman, Bolenge)*

Few women have raised concerns, and this holds true at both sites. Women expected that the local health centre would be extended in the future, with additional rooms for maternal healthcare even an operating theatre, that equipment such as laboratories and ultrasound devices would be added, that medicines would be made more readily available preferably free of charge, as well as an ambulance to transfer women to the referral hospital. They wanted to have a physician as chief of staff at the health centre. The above mentioned expectations were raised mainly by women who had attended health facilities other than the local health centre, or who had heard about other facilities from other persons’ experiences, or who had a history of recent pregnancy complications.

During the interviews, few women in both communities mentioned maternal health services or HPs. Only one woman, belonging to ≤ 19 years group, mentioned poor treatment during delivery. This is in contradiction with key informants who claimed to have heard about many experiences involving inappropriate behaviour and poor treatment in health services but acknowledged that women often did not report these.

### Women’s voice

Regarding concerns or complaints about health services, women need to find ways to communicate these to HPs. In this study, it appeared that women did not express their concerns/complaints to HPs. None of the women interviewed reported having heard about a woman bringing forward her concerns/complaints on her own.

However, it became obvious that when they had concerns/complaints about health services, women often looked for support from their immediate family members especially their husbands, their mothers, and their mothers-in-law. The majority of key informants confirmed that hardly any woman would express concerns and complaints.

### Reported reasons for women not to express concerns and complaints

The researchers explored the reason(s) why this occurred. Apart from the fact that women believed that they were laypersons and therefore unable to judge how health care should be provided, respondents mentioned fear of reprisals as the main reason. Nine respondents from Bolenge and three from Muanda indicated that women also feared that if they expressed their concerns to HPs, the attitude of the latter would change and therefore they may risk poor treatment as illustrated by the following quote:

“Health providers are complicated, if you have a problem with them. They can get angry and abuse you.”

*(Woman, Muanda).*

The majority of respondents at both sites mentioned that there was no formal system in place at the local health centres, or a representative of the population who could present complaints or concerns to HPs. Consequently, women failed to report because they were uncertain on how to voice their concerns, without a risk of reprisal.

Secondly, several respondents from both sites mentioned that women consider themselves as being unable to influence healthcare functioning or the behaviour of HPs, as highlighted by a key informant:

*“*Often, women do nothing; they are disarmed….especially in front of health providers.”

*(KI, Muanda)*

Thirdly, some community members at both sites (*n* = 3) stated that women feared that complaints or concerns would threaten the work of HPs, and that they would be responsible for the “others’ loss of employment” as stated below:

“When I have a complaint about a health care provider, I do not express it because it is not good to put other people’s work at risk. It is his workplace, I cannot endanger his job. Reporting an incident against a provider is considered as endangering his work.”

*(Mother-in-law, Muanda)*

Other reasons mentioned were related to socio-cultural contexts based on age differences. For example, one woman from Bolenge and one from Muanda responded that they were ashamed to report incidents as they were younger than HPs.

Another woman stated that in most Congolese customs, a younger woman is not expected to complain about an older person as highlighted in this quote:

*“I feel deeply ashamed; I do not know how to go and complain to somebody [with laugh]. Health providers are older; I will not feel comfortable to speak to them about my complaints or concerns.“*

*(Woman, Bolenge)*

Three respondents from Bolenge and three from Muanda mentioned that it is not customary for the population to complain, people preferring to wait until the decision maker notices the incident:

*“We do not tell her [the head nurse] anything, we don’t speak, we don’t complain, we are just waiting. We do not know what to do; we are expecting that the authorities themselves will find out.”*

*(Woman, Bolenge)*

Something specific to Muanda: one woman explained that in Muanda, tribesmen prefer not to engage a complaint or a concern in order to avoid trouble in the community.

Another explanation mentioned by three respondents in Muanda and in Bolenge is the preference for the women to rather attend another health facility, using an exit strategy as reported below:

*“*In case of inappropriate practices, it is preferable to go and follow the treatment somewhere else. It can happen that a bad practice occurs once. If for the second time, you come across the same situation, you can go to another healthcare facility.”

*(Man, Muanda)*

### Possible ways to communicate and complain

Reported possibilities to channel concerns and complaints could be regrouped into using; (a) intermediaries, (b) informal communication, and (c) formal structures.

- Using intermediaries

In our study, women answered that they could communicate individually through another person other than the concerned HP. This person could be found within or outside the health centre. For example, five women from Bolenge and eight from Muanda shared that it was possible for them to communicate with; the nurse in charge of the health centre, a Health Zone Officer or another health care provider who may report to the relevant health provider. This depends mainly on the level of confidence the woman has in her relationship with the person she has contacted:

*“When a healthcare provider behaves inappropriately, we prefer to speak to another health worker so that the latter can speak to his colleague. We avoid speaking ourselves in order to avoid problem as the concerned HP could bear a grudge against us and that would become a problem.”*

*(Woman, Muanda)*

Respondents also mentioned that women could use a person outside the health facility; this was often the community health worker (CHW). Eleven respondents from Bolenge and seven from Muanda mentioned that women could report their concerns to CHWs. According to the respondents, CHWs who live in the same community and are involved in health activities within the community could be easily reached and were accessible to the population. This is confirmed by a CHW:

*“The information regarding women’s concerns often stands out when we as CHWs have noticed for example that there is a woman who gave birth in another health facility or at home. We visited her and we asked her why she went elsewhere when there was a health centre there. It is through these visits that we are aware of health providers' absence and a lack of expertise or equipment.*

*We present this to health providers during the Health committee’s meeting”*

*(KI, Muanda)*

Women were said to expect that CHWs could have the courage to talk to HPs, because they were working together. However, two women from Bolenge and one from Muanda expressed doubts about the ability of a CHW to influence the behaviour or decisions of HPs. They did not know what happened after their report to a CHW as reported in this quote:

*“The last time, I told them [CHWs] what I had noticed in the centre. I reported to them because they can have the courage to go to speak to health providers… However, I do not know if they delivered my message.”*

*(Woman, Muanda)*

Community leaders such as a local authority, a village chief, or an administrative chief officer could also be approached. According to three respondents from Bolenge and eight from Muanda, the community leaders could be approached because the women believed that the former could influence the HPs and that their concerns were more likely to be accepted by HPs than when brought by women.

Two community members from Bolenge stated that it could be possible for women to report their concerns or complaints to external persons who could come and ask questions or make surveys in the community for the purpose of collecting population concerns, arguing that these persons could be effective in transmitting these concerns to decision makers. It is interesting to point out that the respondents did not mention national or regional members of parliament even though they have their roots at local level.

(b) Using informal communication

Although people do not complain individually to staff, culturally there are various ways to communicate. For example, eight respondents in Bolenge and two in Muanda mentioned that some women believed that by not approaching anyone to complain, they could express their discontent loudly, through gossip and rustle or create rumours in the community about HPs, hoping that it would reach them.

*“Women like other members of this community always have a behaviour that consists of shouting aloud their complaints about Health Centre on the road. You will see one or two people speak aloud on the road when they disagree…these complaints are often the origin of rumours in the community.”*

*(CHW, Bolenge).*

(c) Using formal structures

According to HPs and some key informants, women could formally report through community health workers to health committees. HPs reported that they used CHWs as means of handling interaction between them and community members and for collecting information from the community and households during home visits. They added that the collected information is presented and discussed during monthly health committee’s meeting as feedback from the community:

*“For example, we learnt that a woman delivered at home through CHWs. As health providers, we know all women who attend antenatal care at the Health Centre, when we realize that a woman does not come any more, we send CHWs to get information about it. So, CHWs came and collected the information regarding what happened and gave us the information.”*

*(Health Provider, Bolenge)*

Two key informants confirmed that which is stated above and added that after the health committee meeting, a report had to be sent to the health zone management team. However, it is important to highlight that community members themselves did not talk about a formal system and that they often talked about CHWs rather than health committees suggesting that most women did not link CHWs to health committees.

Specific to Muanda where Cordaid PBF program was implemented, some key informants (health zone chief officer, purchasing agent manager) mentioned that women could report to members of the community-based organizations when the latter carried out household surveys. According to these key informants, the PBF program included community evaluation of the health centre by community- based organizations that were contracted to make visits in randomly selected community households especially those who had attended the local health centre, for collecting views or experiences about the use of this health centre. The findings of their survey were sent to the health zone management team through the purchasing agent. However, none of the women interviewed mentioned community-based organizations and seemed unaware that there was a local organization that visited community households to collect views or experiences about the use of the local health centre. Furthermore, other key informants and most of the CHWs did not mention having heard about such activities.

It is also worth highlighting that none of the community members interviewed at both sites mentioned that community associations, organizations or coalitions could be used by women to express their voice.

Respondents thought that although there were many associations in their community such as local mutual aid associations, professional associations, nongovernmental organizations, there were no organizations that monitored health centre activities, or had healthcare goals or discussed healthcare issues during their meetings. Furthermore, the women interviewed answered that they did not believe in speaking out in a group or collectively because groups were often not heard by HPs and rulers or that collective action was not appreciated.

“I prefer to go alone to express my concerns. I do not like speaking in a group, because often the group is not well considered….the interlocutor is going to answer you without taking to heart what he tells you. I prefer to express my concerns by myself to be well understood.”

*(Woman, Muanda)*

Moreover, community members did not mention that they could either meet together as a group to present their concerns about healthcare services to the health centre staff, or organize collective actions such as demonstrations, public campaigns, or public hearing meetings.

HPs reported that during health activities such as antenatal care and postnatal care implemented in the health centre, women were given the opportunity to express their concerns:

“During health visits, we allow them to ask questions or voice their concerns, just after the health education session. If a woman has sensitive concerns, she can come to the office, we can discuss them privately.”

(Nurse*, Bolenge)*

HPs mentioned something that was specific to Muanda: when home visits were carried out following up on women who had attended a health centre, the latter were given the opportunity to ask about concerns or complaints.

*“*During home visits I carried out, I ask them [women] questions about nurses’ behaviour during ANC for example. Up to now, they have told us that they are satisfied.”

*(Nurse, Muanda)*

However, health zones chief officers recognized that there is no formal channel that collects information related to the concerns/complaints by the population in the current health information system. They also recognized that most reports from health committees did not mention complaints by the population as well, making it difficult to assess of the responsiveness of healthcare providers to the concerns/complaints of the population. They added that they had never received complaints against healthcare providers via any health committee reports and that not a single health centre offered opportunities to discuss as a team any complaints or concerns by clients.

### Existing community oversight systems

As mentioned above, community members answered that they were excluded in monitoring and evaluating health services. However, according to key informants and HPs, the community exerted oversight on health services through health committees. They reported that during monthly health committee meetings, the nurse- in-charge should provide information about health centre performance to the population through their representatives. During these meetings, community health workers’ representatives should report the information gathered from the community. However, most respondents mentioned that within the health committee the health staff did not share information about health centre performance, but simply shared their expectations from the community in terms of targets and problems regarding health service activities, such as the underachievement of targets regarding the number of antenatal care visits or home deliveries. The interviews by key informants revealed that community health workers’ representatives or committee members participating in these meetings did not deal with community concerns as they did not formally collect them, therefore the health staff was not informed about them. As a consequence, according to the respondents, women were mostly informed about health centre performance or the behaviour of healthcare providers through interpersonal communications rather than through formal systems.

*“*I have no information about the activities of the Health Committee.” (*KI, Muanda)*

*“*The last time we met, I told them [CHW] what I had found in the centre…However, I do not know if they delivered my message.” *(Woman, Muanda)*

*“*Women of these villages, most of the time, do not take a particular action, but they would prefer to go to different health facilities. You will see them taking another direction…they will tell you that they intend to change because some facilities did not exist in the former health centre.” *(CHW, Muanda)*

Another aspect specific to Muanda is the statement made by the manager of the Purchasing Agency that the use of community- based organizations surveys is the community monitoring of health centre performance under PBF settings. The findings of these surveys are reported directly to the Purchasing Agency and not to healthcare providers. It is the Purchasing Agency which presents a summary of the findings during health zone monitoring meetings and provides feedback to health providers. The purchasing agent manager argued that these surveys were aimed at reducing the fear of reprisals and assured more transparency and confidentiality to the community monitoring process. Nevertheless, HPs asserted that because CBOs did not report survey findings to them and that reports were compiled collectively for all health centres belonging to a health zone, they were unaware of grievances brought against them on the one hand. On the other hand, they added that because no community representative participated in health zone monitoring meetings, the community was unaware of either the survey findings of the CBOs or of any decisions made to address them.

### Community enforceability mechanisms

While people do not complain as individuals to staff, culturally there are various ways to acknowledge and communicate good performance and this is mainly done as individuals according to the respondents. For instance, to reward good performance, women thanked health staff, and gave them small gifts or small amounts of money, or even reported positively about HPs. This is embedded in local customs. Women highlighted that it was not compulsory but optional. It is a means for the population to acknowledge what health staff have done for them.

*“The encouragement is often offered individually. For example, when you are satisfied with the service provided, you can willingly reward the health worker with sugar or milk.”*

*(Woman, Bolenge)*

Three community members from Muanda believed that to encourage HPs, women had to continue attending the health centre or to motivate their acquaintances through their testimonies regarding the use of the local health centre instead of going to another health facility. They expected that it would help HPs improve their skills and their income through users’ fees.

Some respondents mentioned that they could report to their acquaintances or members of their social networks, expecting to induce a specific HPs’ behaviour indirectly. For example, five respondents from Muanda and three from Bolenge thought that to discourage improper practices, they should give negative comments about the health centre services to their acquaintances, motivating them to go to another health facility, thus reducing health service intake and affecting either the reputation, the financial turnover or the motivation of HPs.

*“*Women in these villages, most of the time, do not take a particular action, but they would go to a different health facility. You would meet them on the road taking another direction…they would tell you that they were going to a different health facility because of the shortcoming or inadequacies of the health centre.”

*(KI, Muanda)*

On the other hand, this could also work positively: two respondents, a woman and a man, from Muanda mentioned that they could mention to their acquaintances the performance at the health centre, showing the good performance of the health staff. This could reach HPs indirectly. They argued that it was a way of encouraging health staff and attracting more users, thereby increasing a health centre’s financial resources and personnel incentives through users’ fees:

“To encourage them [Healthcare providers], we advertise about their HC. We mentioned their good deeds and give a good report. Good testimonies incite the health staff to work better and maintain an acceptable level of service.”

*(Woman, Muanda)*

Respondents also mentioned that they could discourage malpractices, by thanking or rewarding HPs who performed well in the presence of those who were not aiming to trigger change in the latter. Alternatively, they could do the opposite of what they did to encourage appropriate behaviour or treatment by HPs. However, none of the respondents claimed to have visited traditional healers or traditional birth attendants for reproductive health issues as a consequence of the lack of trust or responsiveness in the health services.

### Health providers’ responsiveness according to the community

Interviews showed that most women and key informants believed that health staff members were responsive to women’s concerns when they talked of their health problems, by supporting their opinions by the perceived attention they received from HPs during health centre visits. They also based their views on the perceived change in the service or HPs’ attitude:

*“When you expressed your concerns, you would find that the staff improved in the next session. For example, a [male] nurse replaced for a time the lady who usually performed Antenatal care. But he was very nervous. I expressed my concerns to the head nurse. The latter talked with him; he changed and became less nervous with clients.”*

*(Woman, Muanda)*

Three respondents from Bolenge and two from Muanda thought that even though there was no change in the provision of health service, the health staff were responsive because they took the time to explain to users why there had been no change or showed a receptive disposition:

*“Health care providers take into account women’s concerns, because they speak about it even if we have not seen clear decisions yet.”*

*(Mother-in-law, Bolenge)*

One community member from Bolenge and two from Muanda stated that HPs were not responsive to patients’ concerns as no change was perceived after receiving those concerns. Moreover, they were likely to get angry and scold the complainant.

## Discussion

In this study we established the extent to which the main elements required for social accountability were perceived and the potential for maternal health services to be strengthened in the DRC. Despite contextual differences, both sites showed similar results. Interestingly, in spite of the low position of the DRC in maternal health statistics [1, 2], women responded to our survey positively about the health care provided. Only a few women voiced explicit complaints against health providers, and some communicated them directly to the health provider. The majority preferred to use indirect channels such as intermediaries, within or outside the health facility, or to go to another health facility. Perceptions on responsiveness of health staff to women’s concerns varied among community members and key informants, and were both positive and negative. This study shows that for community members no formal procedures appeared to be in place to collectively monitor and evaluate health worker performance and to hold health staff accountable. However, two formal routes for accountability seem to exist, according to the healthcare providers or key informants but somehow health providers were not reached. The first being the health committee route where women report their concerns to the community health workers, who in turn report them to a health committee. The second being the CBOs’ route, where women participated in satisfactory surveys carried out by community-based organizations with the results of these surveys being transmitted to health zone meetings and discussed with health providers.

In our study an insignificant number of women expressed their concerns, and this is in sharp contrast to the more critical statements from other community members and the placement of the DRC on the lowest rung of health statistics for maternal health services in the world.

We will discuss below the reason why we regard the self-reported low level of complaints as not being reflective of reality but rather as being caused by context, i.e. unfulfilled preconditions to express complaints. Expressing complaints is an essential first step to let the feedback loop work in the accountability structure. However, complaining presupposes three elements: (i) Knowledge symmetry, women know that the service can and should be better, (ii) Power symmetry, they are able and willing to express their concerns and (iii) Safe conditions, there are opportunities to do so without negative repercussions, e.g., collectively or anonymously.

Low expression of concerns observed in our study could be accounted for through information/knowledge asymmetry. The latter was also observed by Grossmann-Kendall et al. (2001) in Benin, an exploratory qualitative study among 19 women having different backgrounds who had recently given birth in a referral hospital. It was discovered that the majority of women had very little information of medical procedures, causes of complications, purposes of treatments, and hardly had the opportunity to express their views to anyone even to ask a question because of this asymmetry [8]. Similar findings have been reported in other studies and reviews [29–31]. According to Georges (2003), referring to case studies of participatory processes from Asia, especially India, from Europe and from Latin America aiming at improving sexual and reproductive health service delivery and summarizing one of the barriers in accountability mechanisms, people cannot demand services and accountability if they do not know what they are entitled to.

The deficit in expressing concerns may also be associated with illiteracy, education, economic, socio-cultural, reproductive factors, marital status, age, previous health service experiences, and ethnicity. This is in line with other cases from African countries such as Uganda [32], South Africa, Kenya [33] and Malawi, and from Asia (India, Bangladesh, Nepal) and in Latin America (Bolivia). They emphasized the importance of taking into account culture and context when community participation and accountability strategies are to be established and implemented [34, 35].

Secondly, power asymmetry reducing the motivation to speak out was observed in our case and this corroborates studies carried out in Tanzania [5], Benin [29], Uganda [32], and South Africa [33, 36].

For instance, McMahon et al. explore how rural Tanzanian women and their male partners described disrespectful and abusive experiences/treatment during childbirth in facilities and how they responded. They found that, regarding disrespectful and abusive treatment, women were more likely to resign, go back home, reject some facilities and attend different ones. Associated factors reported in existing studies are fear of reprisals, of victimization or further abuse, of not receiving required care, of stigma and shame [30, 31, 37, 38]. D'Oliveira et al. (2002) analysed research from Peru, Brazil, Jamaica, Nigeria, Tanzania, South Africa, Canada, USA, Pakistan, and Turkey and discussed forms of violent abuse by health providers. They related them to the asymmetry of power between health providers and patients and argued that this abuse is a means of controlling patients that is learnt during training and reinforced in health facilities. Power asymmetry can also be rooted in social cultural contexts. Indeed not complaining can be part and parcel of the tribal customs as well as the respect due to older people. Thus power asymmetry is also related to gender relations [10, 38, 39], ethnicity [16, 40, 41], and to the organization and structures within which the individual actor works and lives [42].

Other system factors mentioned are the absence of mechanisms for communicating grievances, the failure of systems to note and punish patient abusers and the indifference of health staff regarding patients complaints, reported in South Africa [36, 43], in Uganda [32] and other countries [33, 37, 38, 44, 45]. There is also a lack of feedback systems in health services [38].

The third element (condition) in citizen engagement encompasses options for collective action to express concerns and anonymous complaints. Our study shows that women did not collectively express concerns, but either refrained from doing anything at all or approached intermediaries such as CHWs, external monitors, authority, and stakeholders in feedback or reporting health care. This choice is based on the women’s perception of the ability of these intermediaries to influence the behaviour of health providers, thus indicating a strategy to counter power asymmetry. The use and importance of intermediaries as brokers between community members and health services have been reported in other studies from Uganda [46], India [47] and also in some review papers [48, 49].

The physical absence of a powerful coalition to voice concerns emerged in our study in spite of the presence of numerous small community associations such as mutual care and support groups, self-help groups, and indigenous community initiatives. This situation hampers social accountability, whereas collective efforts could result in a power balance and at the same time protect individuals who may be put at risk if they contest authorities on their own [45] and offer support to organize public hearings, campaigns or demonstrations related to social pressure and collective rewarding [38]. Moreover, collective action imposes a process of self-reflection among the powerful actors which may result in improvement even though a fundamental shift in the power balance is unnecessary [50]. It is unclear why a stronger coalition to voice concerns did not emerge in our settings. One possible explanation is that in the DRC individual and collective freedoms are not really warranted in practice, and social activities taking collective expressions of opinions are considered as political activities by the rulers. Rulers thus discourage this type of voice, fearing that a community mobilization drives to a broader struggle for democracy and freedom. Also refer to McCoy et al. (2012), who discussed in their systematic literature review an example from apartheid South Africa, where community mobilization around health was a ‘vehicle that helped black communities to develop a sense of control, pride and agency, that were ingredients in the broader struggle for democracy and freedom’ [23].

When women speak out in the hope of receiving the improvements promised, certain procedures to monitor and evaluate the performance of health staff should be in place. Our study highlights that according to women there is a lack of formal community procedures to monitor and evaluate the performance of health staff. In the absence of such formal mechanisms, women can only rely on moral accountability, based on social norms that work through shame and embarrassment, pressures to maintain reputation and status, and the threat of violence. Moral accountability is comparatively less difficult for people to use than engaging in more formally structured means of complaint or feedback [51]. The culture of the Congolese for rewarding and sanctioning individually is based on moral accountability, e.g., by providing allowances or offering grades, provides entry points for more formal mechanisms of social accountability. However, these will need to ‘compete’ with other forms of accountability to be acceptable to health providers. Furthermore rewarding strategies like PBF or community-based health insurance schemes offer an opportunity to further build on.

The absence of a channel to collect information from the population, the perceived ineffectiveness of CHWs and health committees and the failure by health providers to report to the population via health committees [32] limit the responsiveness of the health services. This can be explained by the observation that both the clinical and registration practices in health centres were restricted to the data needed by local health authorities and the Ministry of Health and excluded the experiences and needs of patients and the population. As matter of fact, publishing health centre data as aggregate outcomes [52], can improve awareness and accountability [45]. It is noteworthy that health services are unaware of the concerns of the population and that the population lacks an overview of health service performance.

In this study, we also identified six interesting inconsistencies which might shed light on the complexities behind the causes and solutions to constructively communicate complaints to health providers and actors.

The first contrast being that healthcare providers equate an absence of complaints with an absence of problems, insinuating that they are blameless, even though they are aware that directly communicating a complaint to a health provider is unacceptable, due to gender, knowledge and power asymmetries. The low level of complaint reporting is well documented also in developed countries and should be known by HPs. For instance, Gal and Doron (2007), in a survey carried out in Israel, found that around two-thirds of the persons who had a grievance to report on health service did not complain even though there were formal systems to do so [53]. The situation is exacerbated by the absence or lack of any formal or informal systems of accountability as reported by Goetz [33] in a study carried out in South Africa [36] and which is similar to our case in the DRC. The no complaint = no problem attitude seems to be embedded in the ‘health professional culture that condones controlling behaviour and in ideologies of patients’ inferiority and healthcare providers’ intellectual and moral superiority imparted through training and socialization in health facilities’ [33, 36, 37, 43]. This observation limits the reliability of health providers as informants in accountability mechanisms. Solutions might be found in an extension of the HPs’ curriculum in respect to professional conduct and patient services.

In the second contrast, interviewees in the community stated that there was no formal system for handling complaints or concerns regarding health services while healthcare providers were referring to the health committee and community health workers. This situation can be explained by the fact that community members do not see community health workers as interface actors between health services on the one hand and the community on the other hand, but only as health service agents working within the community as highlighted by Falisse et al. (2012) in a study in Burundi [54] and Bisimwa et al. (2009) in the DRC [55]. Furthermore, community members did not link health committees to CHWs, as also mentioned by Falisse et al. (2012) [54], McCoy et al. (2012) [23], and Goodman et al. (2011) in Kenya [56].

Thirdly, there is an inconsistency between good virtue/intention and good performance. Our study shows that a number of women prefer to see a change in health service after their concerns are raised. For other women an expression of good intention on the part of health workers is acceptable as a response to complaints. This is a phenomenon frequently reported in health system research of people embedded in one historical tradition often failing to see opportunities, which are visible to outsiders. In other words, this passivity stems from health providers who can identify the problem but cannot do anything about it. Furthermore, in most low-income countries confronted by funding constraints, front-line healthcare providers have no control over budget allocation, nor indeed are they able to adequately respond to community expectations. In this situation, the community might value their intention (though incapacitated), rather than their action. This is supported by Berlan and Shiffman (2012) when they reported that consumers view respectful interactions as significant elements of quality care [38]. On the other hand, Bovens (2007) who analysed and assessed accountability, reported that ‘accountability’ is often used interchangeably with virtuous behaviour and is present when public services are performed in a courteous manner [57]. Likewise, in most African norms, an action, even when an action has a good outcome, it is not valued if it is accompanied by a bad attitude. Indeed, even when it has a bad outcome, an action is accepted if it is accompanied by a good attitude/intention. Moreover, adopting a positive attitude towards a woman who is expressing a complaint can be interpreted as an implicit cognitive and silent message as “I received your complaint.” It may also be interpreted as an act of respect towards women’s rights, without changing profoundly the situation of women. That is how providers can keep the power status quo in place.

Fourthly, according to key informants in this study, the PBF survey data were communicated to the health zone level and returned to healthcare providers’ representatives. On the other hand, healthcare providers claimed that they were unaware of any survey results, nor were there any community representatives participating in the meeting when data were shared. This suggests that the data were only openly discussed among health providers, but not across hierarchies. This is due to, the fact that in PBF settings, CBOs are disallowed from to conducting a survey and contacting health providers. This warrants independent and functions separation; and secondly, the results of small surveys are outsourced to purchasing agencies that summarize them for the entire health zone and not for a specific health facility. Therefore, the results are presented as a consolidated situation for all health facilities mostly as comments without compulsion for change. This situation led Falisse et al. (2012) in Burundi to propose that use of CBOs are complementary to health committees for the enhancement of voicing concerns [54].

Fifthly, the study shows that because of power asymmetries, women avoided direct and formal approaches for complaining. At the same time they did not support group or collective action. Women, in our study, asserted that collective action is unwelcomed by local authorities and groups are unheard. All over the world collective action is usually advocated and organized to explicitly express needs and complaints. We surmise that this inconsistency is not only a cultural aspect but is also the result of a political history and of the consideration of people who organized actions in group as ‘political activists.’ Furthermore, they were said to be non-representative of the people on whose behalf they were speaking. According to George (2003), this is one way for authorities to retain power, legitimizing people as beneficiaries and consumers only if they are passive, dependent and isolated individuals, rather than as citizens and active participants in their own health care [45].

This study also highlights solutions to lodge complaints against health providers. Firstly, we mentioned the satisfaction survey conducted by Community Based Organizations (CBOs) under performance-based financing scheme. This allows for avoiding a direct formal approach. Yet, this system is not a channel that people can voluntarily use to voice their concerns unlike other methods described here, but it is a community oversight. Secondly, as reported by Falisse et al. (2012), CBOs are paid for quarterly collecting data from the community, pointing out that financial incentives remain the primary driver of CBOs and not the community interest. Moreover, the users’ information is conveyed only indirectly to health centres and the staff are not compelled to change. Thus the voice of concerns is limited, leaving intact the power of persuasion [54]. However, in the Muanda program, health providers are contractually obliged to make changes in order to maintain the scheme, transforming the Purchasing Agency into a main protagonist of accountability relationships [14]. The CBO survey as community voice remains limited as the population and the community key informants asserted that they ignored them. Thus, in order to strengthen this voice locally, it would be interesting to transmit a copy of their findings to the community through the health committee-community health workers’ network.

Some local actors appear in our study as important players to raise accountability mechanisms. These actors are health services supervisors, local authorities and community health workers. The women interviewed believed that they could influence healthcare providers, who are more likely to accept concerns coming from them. Health supervisors and local authorities are often hierarchically superior to healthcare providers and could be informed indirectly by those who receive reports of misconduct. They are able to act at different levels. For example, the health zone chief officer, being responsible for the health zone, could punish health providers at General Referral Hospitals, either nurses or doctors as well as health centre nurses for their recklessness, when there are complaints against them. Local authorities on their part could counterbalance the health providers’ power at local level, considering that they are traditional power holders and more often members of the health committee. The community health workers representatives are also important players in raising social accountability issues. Unlike common community health workers, these representatives are who for the most part are well educated and more able to counterbalance health providers. The possible implications of those actors were also highlighted by some authors such as Bjorkman and Svensson (2009) in Uganda [52], Maluka (2011) in Tanzania [42] and McCoy et al. (2012).

Our study has some limitations. First, our selection criteria (health zones with active NGOs and functional health centers) introduced a selection bias as the selected zones include high performing health facilities hiding the common DRC health facility situation. However, our selection criteria followed a critical case sampling: if accountability insufficiency related to poor health providers’ attitudes could be found at these particularly well-functioning facilities, then it was likely that the same accountability problems would exist at other health facilities that were less efficient with the current poor functioning regulations for health services. Still, both sites can represent the DRC rural situation, including sites with NGOs supporting the community besides governmental health provisions.

Secondly, limitations are related to data collection. Data were collected from only two health zones and from a small number of respondents, and might not represent the situation in the other zones in the DR Congo. In addition, as data were collected retrospectively there might have been a recall bias. However, in this qualitative study, it is not the representativeness of the sample that matters but the representativeness of information. In our study, we reached saturation and used triangulation to confirm and check the accuracy of data.

Our findings can be comparable to those of countries such as Uganda, Tanzania and India where social accountability was explored considerably, but they are also specific to the DRC. This can suggest that the number of informants was sufficient for collecting the type of issues occurring in the community and that the data are not the result of an unfortunate choice of sites.

Other limitations could be related to research team and reflexivity. The field team consisted of three persons: two female and one male researchers. The two female were single and had not yet experienced either pregnancy or childbirth. Both of them had a social sciences background and had already worked in maternal health research. They were in charge of women’s interviews and were quite similar in terms of age but different with regard to the socio-demographic characteristics (education, occupation, …). The researcher in charge of interviewing key informants, health providers and male community members was a medical doctor, trained in maternal health practice and having quantitative methods background. During his previous experience in health provision, he objectively experienced women health services attendance with its problems such as abusive and disrespectful treatment, delays and women’s mortalities. He and most of the respondents were of the same age. All researchers introduced themselves as research students coming from the local university, the research team members noted their impressions after the interviews, and these were discussed during a daily debriefing meeting. Those notes were included in data analysis. The latter was mainly conducted by the first author. His background could have influenced the data analysis and interpretation. Nevertheless, to reduce these influences, the data analysis was conducted using the SA framework, moreover voice-centered relational method of data analysis was used. This method has reflexive elements built into it. It revolves around a set of three or more readings of the interview text. One of these readings involves a ‘reader- response’ element in which the researcher reads for himself in the text using a ‘worksheet’ technique whereby the respondent’s words are laid out in one column and the researcher’s reactions and interpretations are laid out in an adjacent column [58]. He puts himself, his background, history and experiences in relation to the respondent. He reads the narrative on his own terms, listening for how he is responding emotionally and intellectually to this person. This allows the researcher to examine how and where some of his assumptions and views might affect his interpretation of the respondent’s words, or how he later writes about the person. Finally all the data analysis process was supervised by three supervisors, more experienced in qualitative data analysis.

## Conclusions

This study explores existing mechanisms in rural DRC through which interests of women are expressed and integrated into maternal health service standards in selected sites in DRC. Its findings show that formal social accountability mechanisms are absent in maternal health services in the DRC. Some building blocks which are likely to create social accountability are present. These are community associations and health committees and their interest and willingness to promote collective engagement have to be explored. However, as options for enhancement of voice and oversight, important steps at community and health facility level still have to be taken to allow collective engagement, voice, response and monitoring. It is necessary to ensure that women are aware of their rights to health while at the same time having a safe space to express their concerns when these rights are unmet. Improving social accountability in maternal health services requires intervention by creating spaces where health providers and women are enabled to have constructive dialogue, and health providers can be more receptive to discuss health concerns or demands made by women.

## Additional file

Additional file 1:
**The additional file provides interview guide used showing questions that were asked to respondents.** (DOCX 16 kb)
