# Supplementary material for: Social accountability for maternal health services in Muanda and Bolenge Health Zones, Democratic Republic of Congo: a situation analysis
Source: BMC Health Serv Res. 2015 Nov 23;15:514. doi: 10.1186/s12913-015-1176-6 (PMC4655451; doi:10.1186/s12913-015-1176-6)
Supplement: Additional file 1: — The additional file provides interview guide used showing questions that were asked to respondents. (DOCX 16 kb) [file 12913_2015_1176_MOESM1_ESM.docx]

## Appendix RF2A : Interview guide for with women users and no users

**Study Title: Improving performance and responsiveness of maternal health services through political accountability mechanisms in DR Congo.**

**Research Supervisors: Mambu Nyangi Thérèse, Dieleman Marjolein**

**Student Researcher: Mafuta Eric**

**Performance Sites: Muanda/Bas Congo, Bolenge/Equateur**

**Sponsor: WOTRO program/VU University Amsterdam**

| N° | Themes | Code |
| --- | --- | --- |
|  | INTRODUCTION |  |
|  | We would like first to talk about your experiences with health care during pregnancy or not but related to mother health.   - What type of services do you use as mother or pregnant woman ? |  |
|  | PERFORMANCE OF HEALTH SERVICE |  |
|  | We want to talk about the encounter between the health provider and you during maternal health service attendance in your local health facility:   - Can you talk about your last encounter with the health provider especially about the way you are treated from the moment you arrived to the moment you left *( communication, attention to your word, provider’s behavior during that encounter, Discretion, confidence, Sincerity, Mutual respect)* ? - What did you think about this encounter? Was it positive or negative ?   *( confidence, trust, place, process, hygiene, materials, medicines, schedule)* |  |
|  | We will talk now about the perception of the quality of maternal health services:   - What do you think make the quality of health services? - Do you think that you received quality health care during this last encounter ? *(schedule, effectiveness, distance, cost, beliefs and values, availability, flexibility, agreement, acceptability)* - During your encounter, do you have the impression to be free to choose what service or good you need? Or do you feel pressed by a health provider? - Are you ready to return to attend this health provider or will you be ready to recommend the local health provider to your friend or to your relative? |  |
|  | DELEGATION |  |
|  | - What are your expectations, needs, desires from health care providers? - Did the health services provided meet your expectation ? How (yes) and why (no) ? - What are your ideas how health care should be provided? *What services do you think must be provided ? What services do you think are not provided or are not provided in the way you think it should be provided?* |  |
|  | RESOURCES SUPPLY/FINANCING |  |
|  | You talked about health services you can obtain from health providers.   - Do you think that health providers have resources (technical, financial, materials…) to accomplish their duties, responsibilities or in other words what you need them to do ? if yes, how? If not why? |  |
|  | INFORMATION |  |
|  | We will talk now about the way information from clients and users reach health services providers:   - How do you let health providers know your needs, questions, expectations, opinion or concerns? How do you think this can reach them (systems, mechanism, procedures) ?   *(No feedback from clients, suggestions box, available questionnaire for clients, users’ survey, Official meeting with community leaders, informal discussion with clients or community, protest, feedback, demonstration, coalition, consumers league…..)*   - Do you think that there are persons, individual or groups or organizations that could facilitate you to transmit your view to providers? |  |
|  | RESPONSIVENESS |  |
|  | We will talk about what is done by the health providers to address your need or concerns:   - Did you perceive some changes made by health providers according to the ideas, needs, expectations or concerns expressed by patients? If Yes, Could you indicate what changes were made following what health service area (services, schedule, operations, comfort,…)? - Do you think that health providers value yours views? - Do you feel that it possible for you or the population as a group to ask questions, or to express its concerns , to obtain answers? If yes, how? If no why? |  |
|  | ENFORCEABILITY |  |
|  | We will talk about mechanism or process which can allow clients or their delegate to reinforce or to discourage providers’ behaviors:   - Do you think that health providers are responsible for their behavior; actions and results? - How do you think that you can influence the performance and the behavior of health providers? - What sanctions or penalties do you know to be applied to health providers? *Do you think that there are procedures or mechanisms set up for encouraging good performance or for discouraging the worst ones among health providers?* - Do you know individual or groups or organizations that can allow clients or users to hold health providers responsible *or to make them justify the actions, results or behaviors in front of the community?* |  |
|  |  |  |
|  |  |  |
|  | CONTEXT |  |
|  | We have discussed about mechanisms or process which can allow clients or the representative to hold health providers responsible or accountable for results, actions or behaviors.   - Do you think that there are social or political or local situation or elements from politics, society or others which can make these mechanisms work or not? *Do you think that the context (politics, society, economy or local situation) influence the behavior or the actions of health providers?* |  |
|  | MECHANISMS |  |
|  | We were talking about changes made by health providers according to the ideas or the needs of users. We need to understand why these can occur or why these can not occur ?   - What motivate health providers to meet users’ needs or demands? - What way does voice intervention may work to improve health service delivery? To improve health service uptake by the population? |  |
